# Evaluating the Hydraulic Effects of the Flow through and over the Submerged Biofilter Installed in Polluted Streams

**DOI:** 10.3390/ijerph191610324

**Published:** 2022-08-19

**Authors:** Hany F. Abd-Elhamid, Atef A. El-Saiad, Zeinab I. Salama, Martina Zeleňáková, Emad H. El-Gohary

**Affiliations:** 1 Department of Water and Water Structures Engineering, Faculty of Engineering, Zagazig University, Zagazig 44519, Egypt; 2 Center for Research and Innovation in Construction, Faculty of Civil Engineering, Technical University of Košice, 04200 Košice, Slovakia; 3 Public Directorate for South Sharkia Drainage, Zagazig 44516, Egypt; 4 Institute of Environmental Engineering, Faculty of Civil Engineering, Technical University of Košice, 04200 Košice, Slovakia; 5 Environmental Engineering Department, Faculty of Engineering, Zagazig University, Zagazig 44519, Egypt

**Keywords:** unconventional water resources, water quality, polluted streams, submerged biofilter (SB), hydraulic effects, dimensional analysis

## Abstract

The problem of shortage in freshwater resources in many countries around the world has led to the use of unconventional water resources such as treated wastewater and agricultural drains water to bridge the gap between the demand and supply. However, the open nature of most agricultural drains and the spread of population cumulation around them has made them vulnerable to many organic and inorganic pollutants. One of the artificial methods used to enhance the self-purification process in polluted streams is submerged biofilters (SB). However, most of the previous studies focused on the efficiency of the biofilter to remove the pollutants, and there is a lack of studies on hydraulic changes. This study aims to assess the hydraulic effects of the submerged biofilter of star-shaped plastic media on water streams and develop a mathematical formula that could predict such effects. For this purpose, an experimental study was conducted with 60 total runs (30 for flow through biofilter and 30 for flow over biofilter), and dimensional analyses with multi-linear regression analysis were used to correlate different parameters that affect the flow through and over the biofilter. The mathematical relationships were developed to determine the changes in the upstream water level and that heading up in streams due to the use of the biofilter for both cases of flow. The results of the new formulas are very close to the experimental results, with (R^2^ = 0.89) for flow through the biofilter and (R^2^ = 0.993) for the flow over biofilter. In addition, the results were very close to other developed equations. The developed formulas were used to predict the upstream water depth (h_1_) by knowing the discharge (Q), length (L), and width (B) of the biofilter.

## 1. Introduction

Water is a valuable natural resource, which is an economic good [1] used for different purposes, including drinking, agriculture, fishing, navigation, tourism, and power generation [2]. Water demand has increased because of population growth, agricultural expansion, as well industrial development, and the rise in the standard of living [3]. In fact, the world population is expected to reach over 9 billion in 2050, which will increase the demand for natural resources. Consequently, the global water demand is estimated to increase by 55% in 2050 [4], and according to the Food and Agriculture Organization of the United Nations (FAO), agriculture accounts for approximately 70% of all of the water withdrawn from natural water resources, which is much higher than the amount observed for other sectors (20% industrial and 10% municipal) [5]. Therefore, even if all the industrially and municipally consumed water was reused, it would only cover approximately 43% of the amount needed for agriculture. For this reason, the reuse of wastewater for drinking water or industrial applications is not as cost-effective as it is for irrigation [6]. Since 2005, Egypt has been classified as a water-scarce country, as it has less than 1000 m^3^ of freshwater per year per capita [7], so it has been listed among the ten countries that are threatened by water scarcity in 2025; population predictions will lower Egypt’s per capita share to “absolute water scarcity” of 500 m^3^ per year per capita [8], which is expected to drop to less than 300 m^3^/capita/year in 2050 [9]. Due to the rapid population growth that places Egypt in this dangerous position [10], the total water supply is 74.2 billion m^3^ per year, but the demand will be 75.6 billion m^3^ per year. About 97% of Egypt’s water resources comes from the river Nile, and the rest comes from some rain and non-renewable groundwater aquifers [11]. The main danger for the Nile is that rain fall variability drivers are “found to be the variation in: Sea Surface Temperatures (SST) of the Atlantic and Pacific Oceans (for CEN), sea level pressure of the North Atlantic Ocean and the SST of the Indian Ocean (for SOU), and the SST of Indian and Atlantic Oceans as well as from the Pacific Ocean (for NOR)” [12], which are all beyond the country’s control, and extreme precipitation occurring in the Nile Basin leads to water waste via evaporation [13].

The construction of the Grand Ethiopian Renaissance Dam (GERD), with water storage capacity of 74 billion m^3^, may reduce Egypt’s share of the Nile in the next years. Egypt’s scientists expect a reduction in water share from 20 to 34% due to the construction of GERD. During the dam’s filling period, it was estimated to be between 11 and 19 billion m^3^ [14], and consequently, about one-third of the total agricultural lands might be subjected to drought [15]. In Egypt, The Nile’s water is unsuitable for direct human consumption due to the high level of turbidity, E. coli bacteria, and total coliform and algae that exceed the safety limit of drinking-water standards. The source of the high load of bacteria in the Nile’s water is caused by the domestic raw sewage from agricultural runoff [16]. Generally, rivers and other surface water bodies all over the world are more vulnerable to pollution as compared to groundwater due to direct discharge of untreated industrial, agricultural, and domestic wastewater [17]. With the rise in population, there has been an increase in the quantity of wastes to a proportion that is beyond the limit of the self-purification capacity of water. The accumulation of pollutants in water has resulted in apparent and harmful effects.

Water pollution is also related to the industrialization of civilization and raised living standards, which are directly related to the income of people. Therefore, population growth and industrialization both work synergistically to increase the levels of pollution. The developed countries, having comparatively low populations, are the greatest sufferers of pollution because of their high economic levels, industrialization, and mechanical lifestyle that consumes more resources and energy. On the other hand, the developing countries have overpopulation that increases domestic wastes and sewage. Though these countries do not use much resources and energy, limited finances constrain the treatment of wastes, which are often disposed of untreated, causing severe water pollution problems [18]. The current national plan of Egypt for wastewater treatment, though highly expensive and ambitious, fails in encountering the pressing environmental burdens. This is particularly true in the cities of complex industries, e.g., Ten of Ramadan and northern Cairo. Primary treatment is always insufficient to accomplish the objective for a clean environment. Thus, it is important to adapt inexpensive and simple technology systems regardless of their large area requirement, particularly for sewage treatment [19]. Due to water scarcity in Egypt, agricultural drainage water is commonly reused even though some of these agricultural drains become major carriers of untreated wastewater that is then used for irrigation [20].

The use of submerged biofilters (SB) is one of the proposed methods for improving drain-water quality. Several researchers have offered several techniques for evaluating the use of SB to improve water quality in contaminated drains. Abdel-rahman [21] studied the use of SB in polluted rivers for reducing the organic content. Three different plastic media (plate settler, tube settler, and plastic balls) were used as biofilters. Metcalf and Eddy [22] mentioned that the more common types of anaerobic SBs are upflow packed-bed attached-growth biofilters, upflow attached-growth anaerobic expanded-bed biofilters, attached-growth anaerobic fluidized-bed reactor, and downflow anaerobic attached-growth biofilter. Ramírez-Baca [23] stated that SB is a low-cost system that will enhance the water quality in small, polluted rivers and can be constructed in situ. El Monayeri et al. [24,25,26] investigated the impact of submerged media (pall-rings star-shaped gravel) in four stream pilots on the performance of the biological degradation process and the hydraulic scheme of streams in Egypt. The results indicated that plastic media (pall rings and star shapes) are more effective than gravel in removing BOD and COD despite pall-rings media having the least impact on the hydraulic scheme of the channel’s water flow.

EL-Gohary [27] evaluated the use of submerged biofilters in polluted streams to increase the stream self-purification capacity at the discharging point. He concluded that the performance of submerged biofilters was affected by total hydraulic loading (THL). As the (THL) increases, the COD removal ratio decreases. At the smallest flow rates, the variation of organic volumetric loading rate (VLR) has a slight effect on the performance of biofilters. When compared to star-shaped and gravel biofilters, the pall-rings media biofilter achieved the best COD removal ratio while providing the same total surface area for biofilm formation. When compared to pall-rings and gravel biofilters, the star-shaped media biofilter achieved the highest percent increase in upstream water level and COD-removal ratio. He et al. [28] examined the performance and nitrobacteria population dynamics during the 45-day startup period of a pilot-scale submerged biofilter for landscape river water purification. Salem [29] examined heavy metals and pesticide concentrations in waste water from three major drains in Egypt. The results revealed that quantities of various heavy metals in water samples exceeded the acceptable limits. Pachaiappan et al. [30] provided deep insight into biofiltration technologies engaged in the removal of volatile organic compounds and heavy metals in the wastewater treatment process. The performance of biofiltration relies on important parameters such as filter bed media, microorganisms, temperature, pH, moisture, pressure, and nutrients. Based on the pollutants, these parameters are optimized to obtain high removal efficiency.

As for the hydraulic studies related to the flow through a porous medium (e.g., submerged biofilters), Darcy [31] studied the behavior of flow through porous media considering the flow of water through clean sand filters and proposed that flow of water through a soil could be expressed as [32]:

(1) $$v=k_{c}i=k_{c}\left(\frac{\Delta h}{L}\right)$$

where;

v is the superficial velocity (m/s) or the approach velocity or the discharge velocity;

i is the hydraulic gradient and equals to (Δh/L);

Δh is the head loss over distance (m);

L is the length of filter bed (m);

k_c_ is the coefficient of permeability in (m/s).

Darcy’s law is one of the most important equations in soil mechanics. Equation (1) is usually combined with the continuity equation (Q = Av) and the definition of hydraulic gradient (Δh/L). Darcy’s law is usually written as following [31]:

(2) $$Q=\mathrm{vA}=k_{c}\mathrm{iA}=k_{c}\left(\frac{\Delta h}{L}\right)A$$

where,

Q is the volumetric flow rate (m^3^/s);

A is the cross-sectional area over which flow occurs (m^2^).

The hydraulic conductivity (*K_c_*) is a measure of the ability of water to flow through a porous medium. Holtz [33] mentioned that several factors affect the permeability or hydraulic conductivity of a porous media, including the porosity, the effective grain size, the shape of the voids, flow paths through media voids, and fluid prosperities (viscosity, density, and temperature). El-Gohary [27] used three different media as biofilters, namely pall rings, star shapes, and gravel, for evaluating the effects of the biofilters on heading-up and mentioned that after 50 days of continuous operation with a flow rate of 4.4 L/s, the relative heading-up increased from initial value of 1.128 to 1.232 in the case of gravel media and increased from 1.108 to 1.224 in the case of star-shaped media, while it increased from 1.10 to 1.192 in the case of pall-rings media. Fadhil et al. [34] studied the flow through and over the gravel gabion weirs (GGW) considering the effect of gabion length and height on the upstream water depth in two cases for flow through and transient flow. El-Saiad et al. [35] tested several mathematical equations from the literature to find out the most proper equation that can be used to represent the flow through submerged biofilter. The three equations are described as following:

1. Dupuit formula for flow through horizontal filters
  (3) $$Q=\frac{1.57*B\left(h_{1}^{2}-h_{2}^{2}\right)}{2L}$$
2. Fadhil formula for flow through gravel gabion dams
  (4) $$Q=0.398\frac{h_{1}{}^{4.494}B}{L^{0.193}}$$
3. Fadhil (modified) formula for flow through gravel gabion dams

(5) $$Q=1.64*\frac{{\frac{h_{1}}{h_{2}}}^{12.14}*B}{L^{0.675}}$$

where;

Q is the flow rate (m^3^/s);

B is the width of the biofilter (m);

h_1_ is the depth of water upstream the biofilter (m);

h_2_ is the depth of water downstream the biofilter (m);

L is the length of the biofilter in the flow direction (m).

El-Saiad et al. [35] found that the result of the experimental work is in good agreement with the second equation (Fadhil) and recommended to use it for calculating the flow through the submerged biofilter. Fadhil used gravel media for his biofilter, but El-Saiad et al. [35] used star-shaped plastic media. This study aims to conduct an experimental study for using star-shaped plastic media as a submerged biofilter, measure its effect on the hydraulic properties of the stream, and then develop empirical formulas that represent the flow through and over the submerged biofilter (star shape) based on dimensional analysis and multi regression analysis for the experimental data. According to the authors’ knowledge, no equation has been developed to evaluate the flow through a star-shaped plastic media submerged biofilter. As the relative heading-up of water surface upstream the biofilter is one of the most important variations, the developed formula can be used to evaluate the hydraulic effects of the submerged biofilter on water streams that could predict the heading-up of water surface in streams by knowing the biofilter characteristics, such as the width (B) and length (L) of biofilter and the flow rate (Q) in the stream.

## 2. Materials and Methods

### 2.1. Experimental Work Description

**Figure 1:**
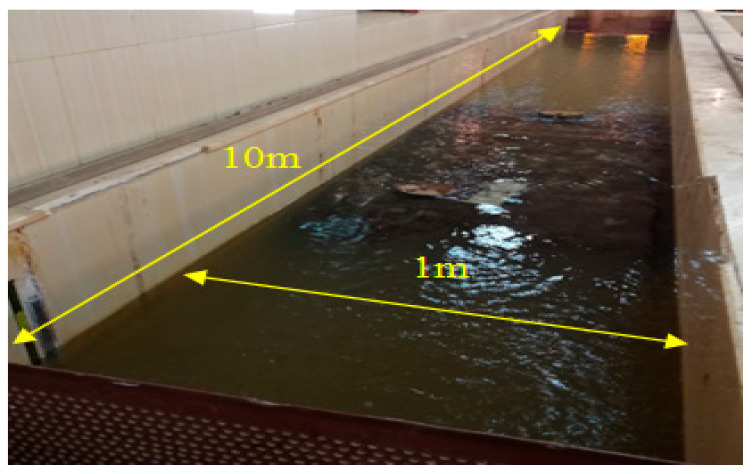
Chanel used in the experimental study.

The experimental work was carried out on a concert channel of 1.0 m width (B), 10 m length, and 1.0 m depth as shown in Figure 1. It has a variable flow rate pump from 2 to 70 L/s and graduated rulers for monitoring water depth upstream and downstream of the horizontal biofilter. The biofilter is made of star-shaped plastic media. The rate of flow in the channel is determined by measuring the head of water (h) above the lab-calibrated weir, which was used to control the inlet discharge to the channel. The discharge equation obtained for the weir can be expressed as [35]:

Q = 0.869 ∗ h^1.69^ (6)

where:

Q is the discharge over crest of the weir in (L/s);

h is the head over the weir crest in (cm).

### 2.2. Biofilter Characteristics

**Figure 2:**
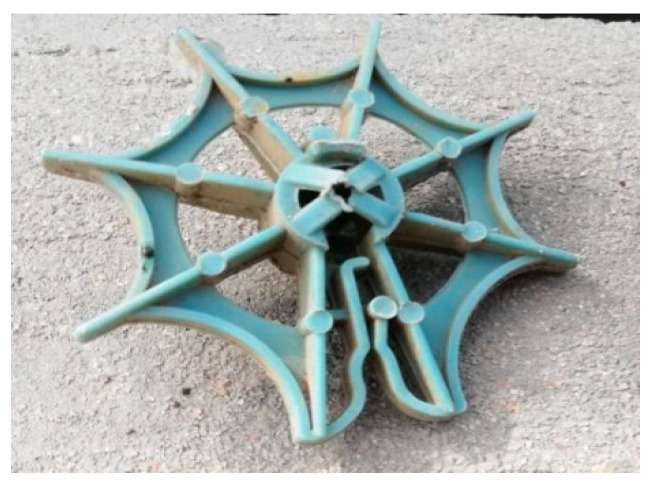
The star-shaped plastic media used as submerged biofilter.

**Figure 3:**
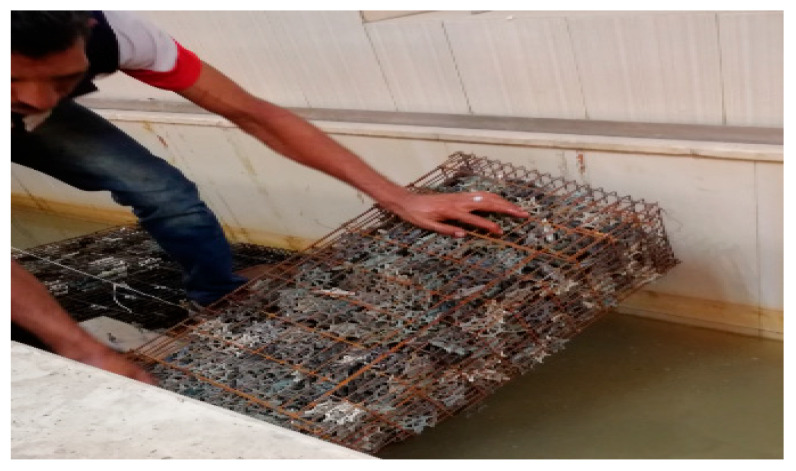
The galvanized steel mesh cages supporting the media installed in the channel.

The media used in this study is a star-shaped plastic media as shown in Figure 2. It was placed in galvanized steel mesh cages of dimensions 1.0 m length, 0.26 m height, and 0.4 m width as shown in Figure 3. Then, it was installed in the stream according to the experimental program. The biofilter performance depends upon the type of media used and its characteristics, such as specific surface area, void ratio, surface roughness, geometry, and configuration. The media used (star-shaped) has a high specific surface area of about 176 m^2^/m^3^ and void ratio of about 87%.

### 2.3. Experimental Program

**Figure 4:**
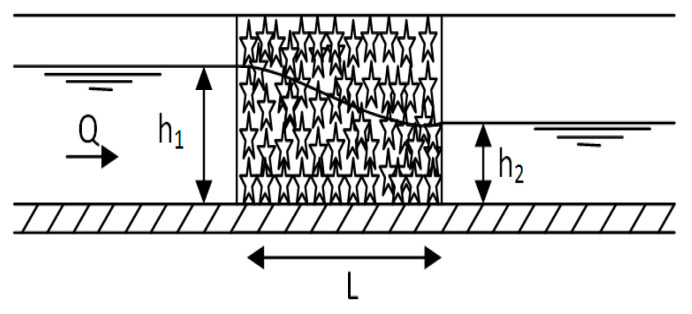
Cross section in the channel where star media was installed.

**Figure 5:**
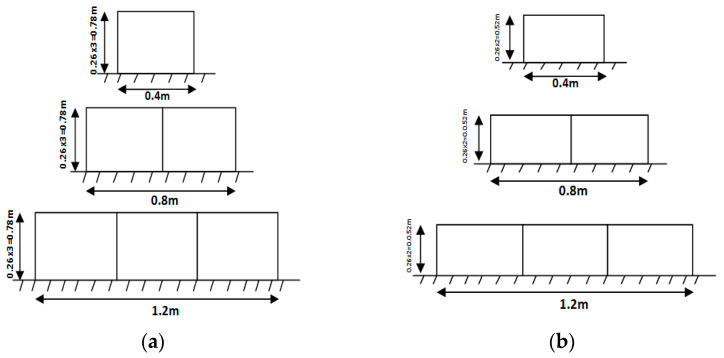
Shape of the biofilter for (**a**) flow through the biofilter and (**b**) flow over the biofilter.

In order to investigate the effect of using submerged biofilter on the relative heading-up, the biofilter length (L) was changed from 0.4 to 1.2 m under variable flow rates for each length as shown in Figure 4. For flow rate, the water depth upstream and downstream the biofilter was measured. The operational conditions applied through the whole study in two cases (flow through the biofilter and flow over the biofilter) are shown in Table 1. The total number of experimental runs or data points was 60 runs for through and overflow experiments and 10 runs for each biofilter length. The experimental work was carried out according to the following steps:

1. The cages containing the plastic media were installed inside the channel crosswise (where the length of the cage is 1.0 m equal to the width of the channel) to achieve a height of 0.78 m in the case of flow through the biofilter and 0.52 during flow over the biofilter (as shown in Figure 5a,b);
2. Establishing a flow rate by adjusting a control valve in the flume supply line;
3. Waiting 10 min so the upstream water depth equilibrium is reached;
4. Recording the discharge and water depth upstream and downstream the filter;
5. Changing the value of the discharge as in step 2 and repeating steps 3 and 4;
6. Repeating steps 2 to 5 for other biofilter length (0.8 m and 1.2 m) 10 times for each length as shown in Figure 5a,b for through flow and overflow regimes.

### 2.4. Measured Upstream and Downstream Water Levels

Table 2 and Table 3 show the obtained results for water depths upstream (h_1_) and downstream (h_2_) and the measured flow rate for flow through biofilter and overflow the biofilter. These results were used to calculate the relative heading-up (h_1_/h_2_), and a new mathematical formula was developed for the determination of the upstream water level due to placing the submerged biofilter in the stream, based on dimensional analysis and multi-regression analysis for two cases.

## 3. Dimensional Analysis and Multi-Linear Regression Analysis

The upstream water depth for the biofilter depends on a number of variables, such as the properties of fluid, media, discharge, and the filter geometry. A physically pertinent relationship between the upstream water depth and other variables may be found by dimensional analysis as in the following.

The functional relationships of the flow rate through the biofilter and over the biofilter (Q) may be expressed as following:

For flow through biofilter

Q = ƒ (ρ, g, B, h_1_, L) (7)

For flow over biofilter

Q = ƒ (ρ, g, B, h_1_, H, L) (8)

in which

Q is the flow rate (m^3^/s);

ρ is the water density (t/m^3^);

g is the gravitational acceleration (m/s^2^);

h_1_ is the depth of water upstream the biofilter (m);

B is the width of the biofilter (m);

H is the height of biofilter (m);

L is the length of the biofilter in the flow direction (m).

Based on the flow through and over the biofilter, depending on the relationships 7 and 8, some transformations lead to the non-dimensional relationships 9 and 10.

For flow through biofilter

(9) $$\frac{Q}{g^{0.5}h^{2.5}}=\Phi \left(\frac{B}{h},\frac{L}{h}\right)$$

For flow over biofilter

(10) $$\frac{Q}{g^{0.5}h^{2.5}}=\Phi \left(\frac{B}{h},\frac{L}{h},\frac{H}{h}\right)$$

Then, the dimensionless group in the relationships 9 and 10 is correlated to give an explicit equation for computing flow rate(Q) through and above the biofilter. A multi-linear regression analysis is used to correlate the different dimensionless parameters shown in Equations (7) and (8) to develop an empirical equation that connecting the flow rate through the biofilter (Q) to the biofilter length (L), width (B), and upstream water depth (h_1_) as shown in Equation (11) and connecting the flow over the biofilter (Q) to the biofilter length (L), width (B), and upstream water depth (h_1_), where biofilter height was used because it was constant in this study as shown in Equation (12). The flow rate is expressed as following in two cases:

For flow through biofilter

(11) $$Q=0.7487\frac{h_{1}{}^{5.734}B}{L^{0.134}}$$

For flow over biofilter

(12) $$Q=1.62\frac{h_{1}{}^{6.79}B}{L^{0.0826}}$$

in which

Q is the flow rate (m^3^/s);

h_1_ is the depth of water upstream the biofilter (m);

B is the width of the biofilter (m);

L is the length of the biofilter in the flow direction (m).

The formula developed by multi-linear regression analyses 11 and 12 can be used to obtain the upstream water depth (h_1_) as follows:

For flow through biofilter

(13) $$h_{1}=1.052\frac{Q^{0.1744}L^{0.0233}}{B^{0.1744}}$$

For flow over biofilter

(14) $$h_{1}=0.932{\;*\;L}^{0.0122}*{\left(\frac{Q}{B}\right)}^{0.1473}$$

## 4. Result and Discussion

The measured flow rate and the resulting relative heading-up (h_1_/h_2_) of the experimental study for 30 runs are shown in Table 4. The flow rate is calculated using two equations from the literature (Dupuit formula, Fadhil equation) and compared with the flow rate calculated from the developed empirical Equation (11) for the flow through the biofilter. The results showed that, for each length of biofilter, when increasing the discharge, the relative heading-up increases. The biofilter length L = 1.2 m caused the highest values of relative heading-up, which ranged from 1.02 to 1.13 when increasing the flow rates from 20.6 to 64.6 L/s; followed by L = 0.8 m, for which the relative heading-up increased from 1.01 to 1.095; and L = 0.4 m, for which the relative heading up increased from 1.002 to 1.048 as shown in Table 2. Table 4 shows the result of calculated flow rates passing through the biofilter using the new empirical Formula (11) compared to the calculated flow rate by the Dupuis (3) and Fadhil (4) equations. The developed equation gave correlation coefficient (R^2^ = 0.89), which is closer to values determined by the Fadhil equation than the Dupuit equation. It can be concluded that the flow rate passing through the biofilter depends on the upstream water level (h_1_), the length (L), and width (B) of the biofilter.

The measured flow rate and the resulting relative heading-up (h_1_/h_2_) of the experimental study for 30 runs for flow over biofilter is shown in Table 5. The flow rate is calculated using the Fadhil Equation (4) from the literature compared with the flow rate calculated from the developed empirical Equation (12). The results showed that, for each length of biofilter, when increasing the discharge, the relative heading-up increases. When the flow rates increased from 18.7 to 64.2 L/s, the biofilter length L = 1.2 m caused the highest values of relative heading-up, which ranged from 1.25 to 1.040; followed by L = 0.8 m, for which the relative heading up increased from 1.020 to 1.035; and L = 0.4 m, for which the relative heading-up increased from 1.014 to 1.025 as shown in Table 3.

**Figure 6:**
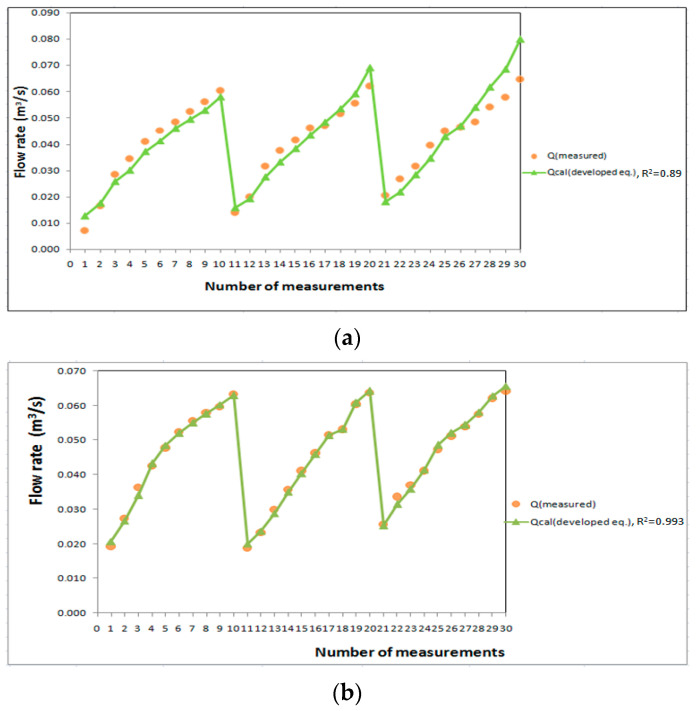
Comparison between the flow rate through the biofilter by the developed formula with the measured values for (**a**) flow through the biofilter and (**b**) flow over the biofilter.

The results of the experimental study were compared with the flow rate obtained from the developed Formulas (11) and (12) for flow through and over the biofilter as shown in Figure 6. The developed formula for the flow through the submerged biofilter was used to improve polluted water streams with the correlation coefficient R^2^ = 0.89 for flow through biofilter as shown in Figure 6a and R^2^ = 0.993 for flow over biofilter as shown in Figure 6b. It is clear that the flow rate passing through the biofilter depends on the height of the water in front of it (h_1_) more than on the relative heading-up (h_1_/h_2_). The results of the developed equations are in good agreement with the experimental results.

**Figure 7:**
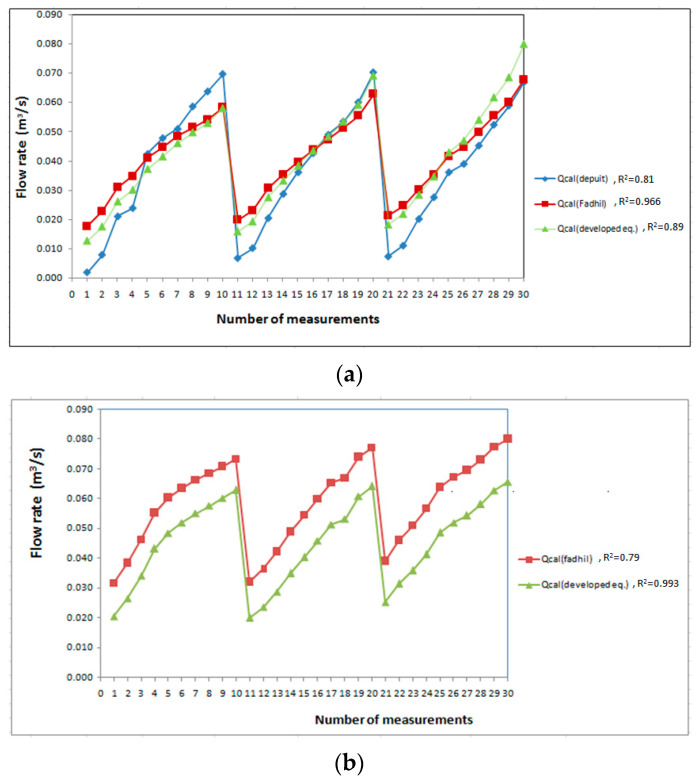
The flow rate through the biofilter by the developed formula compared with Depuit and Fadhil: (**a**) flow through the biofilter and (**b**) flow over the biofilter.

Furthermore, the results of the developed Equations (11) and (12) were compared with the flow rate obtained from the Dupuit and Fadhil equations for flow through the biofilter, and we compared with the Fadhil equation over the the flow above the biofilter as shown in Figure 7. The results showed that the flow rate of the developed formula for the flow through the biofilter is closer to the Fahil equation than the Depuit as shown in Figure 7a,b. Dupuit presented only one equation for flow throgh the biofilter but Fadhil presented two equations for both flow through and above the biofilter of gravel media. The results of the developed equations are in good agreement with the other equations results for both cases of flow through and above the biofilter.

**Figure 8:**
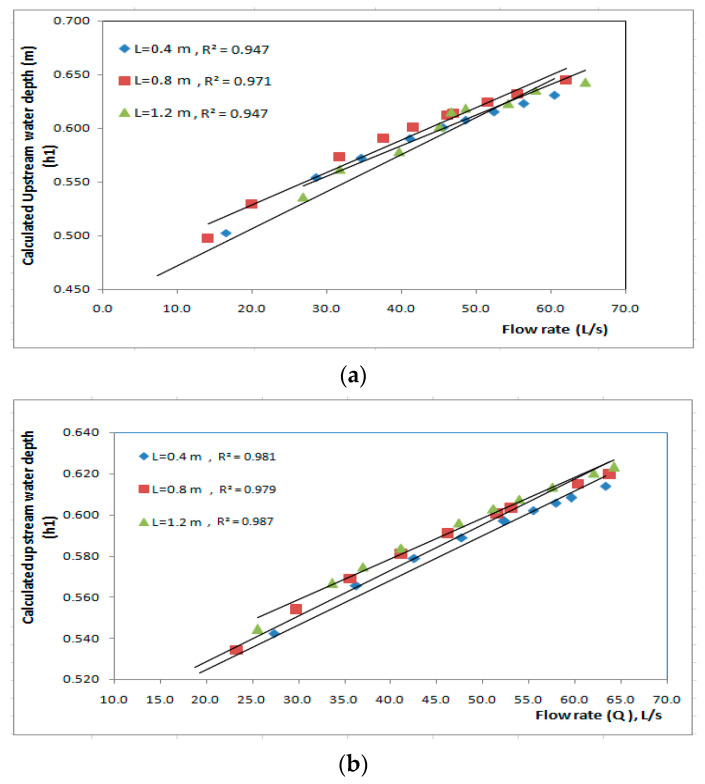
Calculated values of upstream water depths for different lengths of the biofilter at different flow rates for (**a**) flow through the biofilter and (**b**) flow over the biofilter.

Equations (13) and (14) are used to determine the upstream water level (h_1_) at different lengths for different discharge. Figure 8a shows the relationship between calculated upstream water depth (h_1_) and discharge (Q) passing through the biofilter at different length of biofilter (0.4 m, 0.8 m, and 1.2 m). The relationship between calculated upstream water depth (h_1_) and discharge (Q) is linear for all the lengths of biofilter. For same discharge, the upstream water depth (h_1_) value increased with increasing length of the biofilter. As shown in Figure 8a, the length L = 1.2 m gave the highest values of calculated upstream water depth (h_1_), which ranged from 0.436 m to 0.631 m when the flow rates increased from 7.2 to 60.4 L/s with correlation coefficient R^2^ = 0.947, followed by the length L = 0.8 m, for which the upstream water depth (h_1_) ranged from 0.498 to 0.644 m when the flow rate increased from 14.1 to 62.1 L/s with correlation coefficient R^2^ = 0.971. However, the length L = 0.4 m gave the lowest values of the upstream water depth (h_1_), which ranged from 0.537 m to 0.655 m when the flow rates increased from 20.6 to 64.6 L/s with correlation coefficient R^2^ = 0.947. The same was shown for flow over the biofilter as shown in Figure 8b.

**Figure 9:**
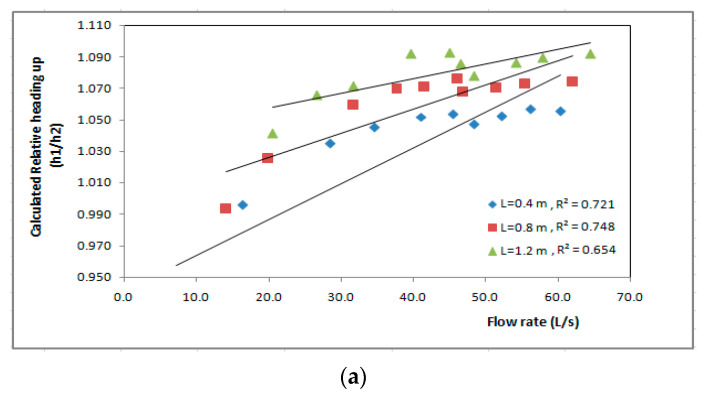

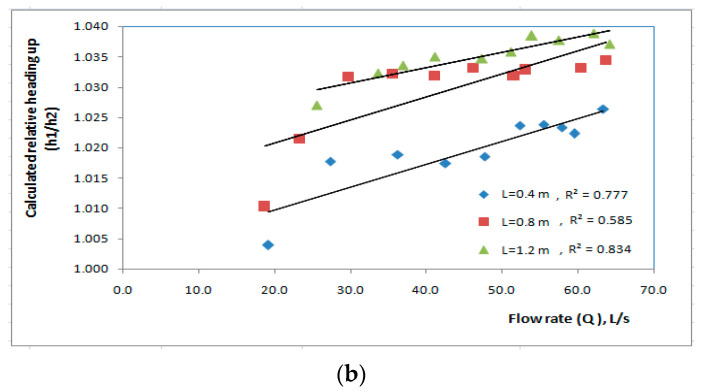
Calculated values of relative heading-up for different lengths of the biofilter at different flow rates for (**a**) flow through the biofilter and (**b**) flow over the biofilter.

For flow through and over the biofilter, the relationship between calculated relative heading-up (h_1_/h_2_) and discharge (Q) is linear for all the lengths of biofilter. For same discharge, the calculated relative heading-up (h_1_/h_2_) value increased with increasing length of the biofilter. As shown in Figure 9, the length L = 1.2 m gave the highest values of calculated relative heading-up with correlation coefficient R^2^ = 0.721, 0.777, respectively, followed by the length L = 0.8 m with correlation coefficient R^2^ = 0.748, 0.585, respectively. However, the length L = 0.4 m gave the lowest values of the calculated relative heading-up with correlation coefficient R^2^ = 0.654, 0.834, respectively, as shown in Figure 9a, and the same was shown for flow over the biofilter as shown in Figure 9b.

**Figure 10:**
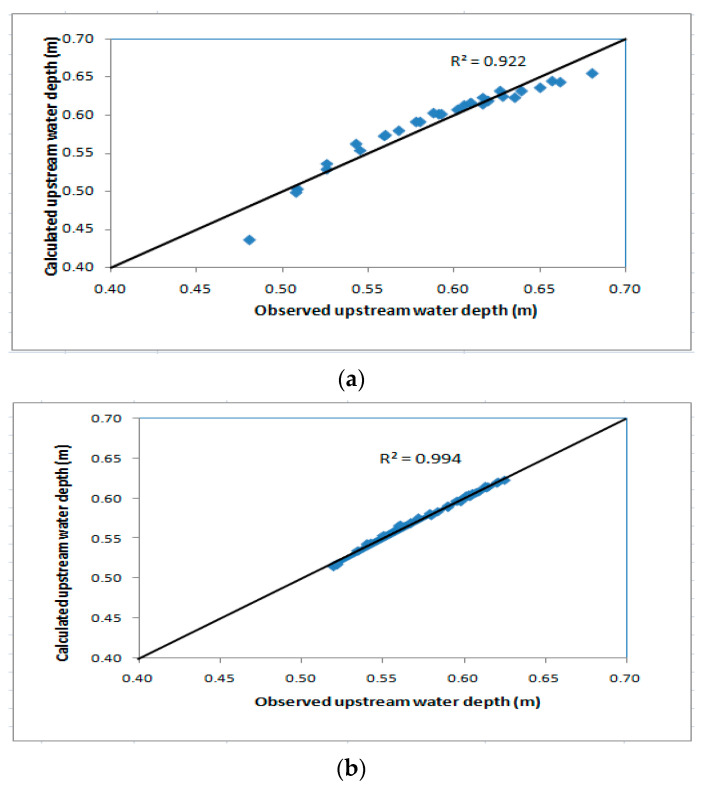
Calculated upstream water depths against measured upstream water depths for (**a**) flow through the biofilter and (**b**) flow over the biofilter.

The results of calculated upstream water depths (h_1_) using Equation (13) are plotted against the measured values in the laboratory for flow through biofilter. A good agreement was obtained between the calculated upstream water depth (h_1_) and the measured in the laboratory as shown in Figure 10a, and the same was obtained for flow over the biofilter as shown in Figure 10b.

Multiple regression analysis based on the dimensional analysis concept was used to create new formulas for determining the upstream water depth for flow through and over the biofilter of star-shaped plastic media. The equations correlated the upstream water depth with the length and width of the biofilter and flow rate through the filter. The used media (star-shaped) is cheap and available compared to other types of media [26]. The developed equation can be used to calculate the upstream water depth for different biofilter lengths. This could help in optimizing the biofilter dimensions to minimize the upstream water that affects the heading-up. Using optimization techniques is recommended for future studies to minimize the biofilter dimensions to reduce the heading-up due to the installation of biofilter in polluted streams. However, some other parameters may be measured, such as velocity, which can be used to calculate the Reynolds number, that can be considered in future work.

## 5. Conclusions

Water scarcity is considered the major challenge that faces many countries around the world. It is an imbalance condition that occurs due to the lack of freshwater resources and increasing water demand. Hence, the Egyptian government is focusing on reusing treated wastewater to substitute the shortage of water resources. Treated wastewater (TWW) is a reliable water source that can fill the gap between water demand and supply. Submerged biofilters (SB) is one of the artificial methods that can be used to enhance the self-purification process in polluted streams. In this study, an experimental work was caried out to evaluate the hydraulic effects of biofilter on the polluted stream. Based on the experimental work done for star-shaped biofilter, it was found that the relationship between relative heading-up and discharge through the biofilter is linear for all biofilter lengths. It was observed that increasing the biofilter length increases the relative heading-up. The biofilter of length L = 1.2 m caused the highest values of relative heading-up, which ranged from 1.02 to 1.13 when increasing the flow rates from 20.6 to 64.6 L/s; followed by length L = 0.8 m, which increased the relative heading-up from 1.01 to 1.095; and the length L = 0.4 m, which increased the relative heading-up from 1.002 to 1.048. Dimensional analysis and multi-linear regression analysis were used to develop two new empirical formulas for calculating flow rate through and over the star-shaped biofilter based on the experimental data. The new empirical formulas are very close to values determined experimentally with (R^2^ = 0.89) for flow through the biofilter and (R^2^ = 0.993) for the flow over biofilter. The flow rate passing through and over the biofilter depends on the height of the water in the upstream (h_1_), the length (L), and width (B) of the used biofilter. The developed formulas were used to predict the upstream water depth (h_1_) by knowing the discharge (Q), length (L), and width (B) of the biofilter and controlling it before using biofilter. The developed equations can help in predicting the hydraulic effects of using submerged biofilter in polluted streams, which can be used for enhancing the polluted stream characteristics. Optimization techniques could help in minimizing the biofilter dimensions to reduce the heading-up, which could increase the efficiency of installation of biofilters in polluted streams.

## Figures and Tables

**Table 1:** The operational conditions applied through the whole study.

Runs	Case of Flow	Length (m)	Flow Rate (L/S)	Measured Parameters	Calculated Parameters
1	Flow through the biofilter	0.4	7.2 to 60.4	h_1_, h_2_	Relative heading-up (h_1_/h_2_)
2		0.8	14.1 to 62.1		
3		1.2	20.6 to 64.6		
1	Flow over the biofilter	0.4	19.2 to 63.3		
2		0.8	18.7 to 63.7		
3		1.2	18.5 to 64.2		

**Table 2:** The measured flow rate, water depths upstream and downstream, and relative heading-up for different length of the biofilter (case of flow through the biofilter).

No	L (m)	Measured Flow Rate (m^3^/s)	Upstream Water Depth (h_1_) m	Downstream Water Depth (h_2_) m	Relative Heading-Up (h_1_/h_2_)
1	0.4	0.007	0.481	0.480	1.002
2		0.016	0.509	0.505	1.008
3		0.029	0.545	0.535	1.019
4		0.035	0.559	0.548	1.020
5		0.041	0.580	0.561	1.034
6		0.045	0.591	0.570	1.037
7		0.048	0.602	0.580	1.038
8		0.052	0.610	0.585	1.043
9		0.056	0.617	0.590	1.046
10		0.060	0.627	0.598	1.048
11	0.8	0.014	0.508	0.501	1.014
12		0.020	0.526	0.516	1.019
13		0.032	0.560	0.541	1.035
14		0.038	0.578	0.552	1.047
15		0.041	0.593	0.561	1.057
16		0.046	0.606	0.569	1.065
17		0.047	0.617	0.575	1.073
18		0.052	0.628	0.583	1.077
19		0.055	0.639	0.589	1.085
20		0.062	0.657	0.600	1.095
21	1.2	0.021	0.526	0.515	1.021
22		0.027	0.543	0.527	1.030
23		0.032	0.568	0.540	1.052
24		0.040	0.588	0.551	1.067
25		0.045	0.610	0.563	1.083
26		0.047	0.620	0.570	1.088
27		0.048	0.635	0.578	1.099
28		0.054	0.650	0.585	1.111
29		0.058	0.662	0.590	1.122
30		0.065	0.680	0.600	1.133

**Table 3:** The measured flow rate, water depths upstream and downstream, and relative heading-up for different length of the biofilter (case of flow over the biofilter).

No	L (m)	Measured Flow Rate (m^3^/s)	Upstream Water Depth (h_1_) m	Downstream Water Depth (h_2_) m	Relative Heading Up (h_1_/h_2_)
1	0.4	0.019	0.52	0.513	1.014
2		0.027	0.54	0.533	1.013
3		0.036	0.56	0.555	1.009
4		0.043	0.58	0.569	1.019
5		0.048	0.59	0.578	1.021
6		0.052	0.596	0.583	1.022
7		0.055	0.601	0.588	1.022
8		0.058	0.605	0.592	1.022
9		0.060	0.609	0.595	1.024
10		0.063	0.613	0.598	1.025
11	0.8	0.019	0.522	0.512	1.020
12		0.023	0.535	0.523	1.023
13		0.030	0.551	0.537	1.026
14		0.036	0.567	0.551	1.029
15		0.041	0.579	0.563	1.028
16		0.046	0.59	0.572	1.031
17		0.052	0.60	0.582	1.031
18		0.053	0.603	0.584	1.033
19		0.060	0.615	0.595	1.034
20		0.064	0.62	0.599	1.035
21	1.2	0.026	0.543	0.530	1.025
22		0.034	0.561	0.549	1.022
23		0.037	0.572	0.556	1.029
24		0.041	0.584	0.564	1.035
25		0.047	0.598	0.576	1.038
26		0.051	0.604	0.582	1.038
27		0.054	0.608	0.585	1.039
28		0.058	0.614	0.591	1.039
29		0.062	0.621	0.597	1.040
30		0.064	0.625	0.601	1.040

**Table 4:** Calculated flow rate by Dupuit, Fadhil, and the developed equation and the resulting upstream water level (h_1_) and relative heading-up (h_1_/h_2_) for flow through the biofilter.

No.	L (m)	Measured Flow Rate (m^3^/s)	Calculated Flow Rate Q(m^3^/s)			Calculated Upstream Water Level (h_1_) m (Developed eq.)	Calculated Relative Heading-Up (h_1_/h_2_)
			Dupuit	Fadhil	Developed Equation		
1	0.4	0.007	0.002	0.018	0.013	0.436	0.908
2		0.016	0.008	0.023	0.018	0.503	0.996
3		0.029	0.021	0.031	0.026	0.554	1.035
4		0.035	0.024	0.035	0.030	0.573	1.045
5		0.041	0.043	0.041	0.037	0.590	1.052
6		0.045	0.048	0.045	0.041	0.601	1.054
7		0.048	0.051	0.049	0.046	0.607	1.047
8		0.052	0.059	0.052	0.050	0.616	1.052
9		0.056	0.064	0.054	0.053	0.623	1.057
10		0.060	0.070	0.058	0.058	0.631	1.055
11	0.8	0.014	0.007	0.020	0.016	0.498	0.993
12		0.020	0.010	0.023	0.019	0.529	1.025
13		0.032	0.021	0.031	0.028	0.573	1.060
14		0.038	0.029	0.035	0.033	0.591	1.070
15		0.041	0.036	0.040	0.039	0.601	1.071
16		0.046	0.043	0.044	0.044	0.612	1.076
17		0.047	0.049	0.047	0.048	0.614	1.068
18		0.052	0.053	0.051	0.054	0.624	1.070
19		0.055	0.060	0.056	0.059	0.632	1.073
20		0.062	0.070	0.063	0.069	0.644	1.074
21	1.2	0.021	0.007	0.021	0.018	0.537	1.042
22		0.027	0.011	0.025	0.022	0.562	1.066
23		0.032	0.020	0.030	0.029	0.579	1.072
24		0.040	0.028	0.035	0.035	0.602	1.092
25		0.045	0.036	0.042	0.043	0.615	1.093
26		0.047	0.039	0.045	0.047	0.619	1.086
27		0.048	0.045	0.050	0.054	0.623	1.078
28		0.054	0.053	0.055	0.062	0.636	1.087
29		0.058	0.059	0.060	0.069	0.643	1.090
30		0.065	0.067	0.068	0.080	0.655	1.092

**Table 5:** Calculated flow rate by Fadhil and the developed equation and the resulting upstream water level (h_1_) and relative heading-up (h_1_/h_2_) for flow over the biofilter.

No.	L (m)	Measured Flow Rate (m^3^/s)	Calculated Flow Rate Q(m^3^/s)		Calculated Upstream Water Level (h_1_) m	Calculated Relative Heading-Up (h_1_/h_2_)
			Fadhil	Developed Equation		
1	0.4	0.019	0.032	0.021	0.515	1.004
2		0.027	0.038	0.027	0.542	1.018
3		0.036	0.046	0.034	0.565	1.019
4		0.043	0.055	0.043	0.579	1.017
5		0.048	0.060	0.049	0.589	1.019
6		0.052	0.064	0.052	0.597	1.024
7		0.055	0.066	0.055	0.602	1.024
8		0.058	0.069	0.058	0.606	1.023
9		0.060	0.071	0.060	0.608	1.022
10		0.063	0.073	0.063	0.614	1.026
11	0.8	0.019	0.032	0.020	0.517	1.010
12		0.023	0.036	0.024	0.534	1.021
13		0.030	0.042	0.029	0.554	1.032
14		0.036	0.049	0.035	0.569	1.032
15		0.041	0.054	0.040	0.581	1.032
16		0.046	0.060	0.046	0.591	1.033
17		0.052	0.065	0.051	0.601	1.032
18		0.053	0.067	0.053	0.603	1.033
19		0.060	0.074	0.061	0.615	1.033
20		0.064	0.077	0.064	0.620	1.034
21	1.2	0.026	0.039	0.025	0.544	1.027
22		0.034	0.046	0.031	0.567	1.032
23		0.037	0.051	0.036	0.575	1.034
24		0.041	0.057	0.041	0.584	1.035
25		0.047	0.064	0.049	0.596	1.035
26		0.051	0.067	0.052	0.603	1.036
27		0.054	0.070	0.054	0.608	1.038
28		0.058	0.073	0.058	0.613	1.038
29		0.062	0.077	0.063	0.620	1.039
30		0.064	0.080	0.066	0.623	1.037

## Data Availability

Not applicable.
